# The Many Faces of Mitochondrial Autophagy: Making Sense of Contrasting Observations in Recent Research

**DOI:** 10.1155/2012/431684

**Published:** 2012-04-08

**Authors:** Alexander I. May, Rodney J. Devenish, Mark Prescott

**Affiliations:** Department of Biochemistry and Molecular Biology, Monash University, Clayton Campus, Clayton VIC 3800, Australia

## Abstract

Research into the selective autophagic degradation of mitochondria—mitophagy—has intensified in recent years, yielding significant insights into the function, mechanism, and regulation of this process in the eukaryotic cell. However, while some molecular players in budding yeast, such as Atg32p, Uth1p, and Aup1p, have been identified, studies further interrogating the mechanistic and regulatory features of mitophagy have yielded inconsistent and sometimes conflicting results. In this review, we focus on the current understanding of mitophagy mechanism, induction, and regulation in yeast, and suggest that differences in experimental conditions used in the various studies of mitophagy may contribute to the observed discrepancies. Consideration and understanding of these differences may help place the mechanism and regulation of mitophagy in context, and further indicate the intricate role that this essential process plays in the life and death of eukaryotic cells.

## 1. Introduction

Even within large multicellular organisms, cells are not guaranteed a life within completely stable tissue environments. The ability of cells to adapt to stressful conditions, such as nutrient limitation, is a fundamental homeostatic requirement in order to survive and proliferate. The highly conserved process of autophagy is an important adaptation to the diverse challenges presented by environments in which unicellular and multicellular eukaryotic cells exist. Essentially, this process involves the transport of cellular components to the lysosome (in mammals) or vacuole (yeast) for degradation to fundamental components that are then recycled by the cell. In recent years, both nonselective and selective forms of autophagy, the uptake of bulk, random portions of the cytosol, or of specific targets, respectively, have been described.

Targets of the selective autophagic machinery include organelles, protein aggregates, and even invading microorganisms. Mitochondria, which accrue damage as they age, can present a challenge to the cell through uncontrolled generation of reactive oxygen species (ROS) and become increasingly inefficient in their generation of ATP. The selective removal of mitochondria by autophagy, known as mitophagy, is an important cellular adaptation to the challenge presented by this important organelle and the potential hazard it represents. Recently, studies have revealed key proteins involved in mitophagy, providing insights into the mechanism of this process. As the focus of research increasingly falls upon the physiological role of mitophagy within the cell, it is important to consider the wider meaning of results obtained to date in order to better understand the physiological role of mitophagy.

In this review, we briefly overview the current understanding of the mechanism of mitophagy, focussing on the model organism of the field, the budding yeast *Saccharomyces cerevisiae*. Stressors known to induce mitophagy in yeast will then be discussed, before recent research interrogating the regulation of mitochondrial turnover is addressed. Finally, discrepancies apparent in research undertaken to date will be addressed with reference to the experimental conditions employed in these studies and their relationship to our current understanding of mitophagy.

## 2. Autophagy and Mitophagy as Unique Forms of Intracellular Degradation

**Figure 1:**
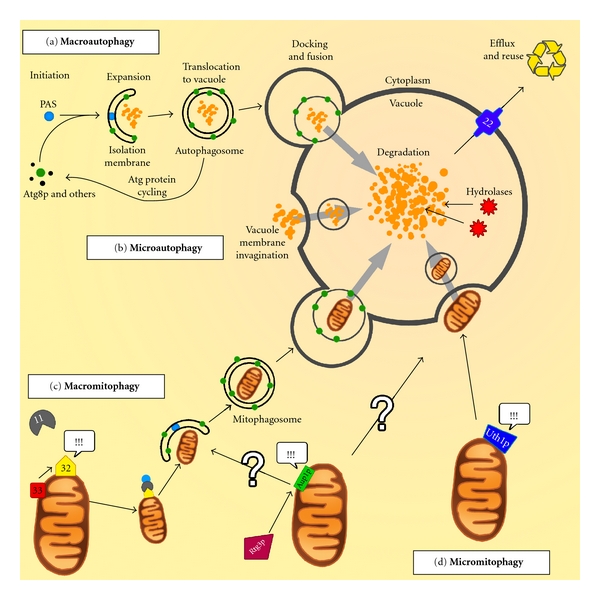
Overview of autophagy and mitophagy in yeast. (a) Macroautophagy, through the Atg proteins (including Atg8p, green dots), sequesters cytoplasmic components into autophagosomes for delivery to the vacuole for degradation. (b) Microautophagy involves invagination of the vacuolar membrane in order to take up cytoplasmic contents for degradation. (c) Mitochondria can be selectively degraded through a microautophagic mechanism. This requires the activity of Atg32p, Atg33p and Atg11p to bring the selected mitochondria into contact with the core autophagy machinery. (d) Mitochondria can also be removed by selective microautophagy, or micromitophagy, the mechanism of which remains unclear. While Atg32p, and Atg11p may be involved in micromitophagy, there is no definitive evidence to support this and the mechanism of Aup1p and Rtg3p function remains undetermined. See text for details. !!! = Inducing signal, ? = Uncertain mechanism.

Macroautophagy (usually referred to as *autophagy*) involves the sequestration of cytoplasmic components (ranging from protein aggregates to whole organelles) into double-membrane structures known as autophagosomes (APs) (Figure 1(a)) [1]. Autophagosomes are delivered to the cell's degradative compartment, the vacuole (lysosomes in mammals), their contents degraded and subsequently returned to the cytoplasm for reuse. This important ability to “recycle” dangerous or unnecessary parts of the cell, described in numerous reviews [2–4], provides components during times of stress, allowing the cell to fulfil essential metabolic requirements [5]. However, autophagy also plays an important role in cellular homoeostasis, and a basal level of autophagy is evident in eukaryotic cells as a fundamental degradation pathway [6].

In the yeast *Saccharomyces cerevisiae*, the autophagy-related (*ATG*) genes encode proteins involved in autophagy, with 31 of these identified so far. Proteins implicated in all autophagic processes, encoded by the “core” *ATG* genes, constitute the basic autophagy machinery [7]. Homologues of many of these proteins have been identified in mammalian cells, demonstrating the highly conserved nature of autophagy throughout eukaryotic organisms [4]. While the process in yeast is described in detail elsewhere [3, 7, 8], it is useful to briefly consider key features of autophagy.

In yeast, a collection of 16 core Atg proteins are involved in the formation of the preautophagosomal structure (PAS), a transiently formed nucleation site [7]. From the PAS, membranes are recruited from a source that remains controversial; studies in yeast and mammalian cells variously suggest the plasma membrane [9], Golgi apparatus [10], endoplasmic reticulum [11], and the mitochondrion [12] as the source of membranes for AP expansion. As the AP expands, forming a double-membrane structure, it captures a portion of the cytosol destined for degradation [13]. The completed AP then traffics to the vacuole, where its outer membrane fuses with the vacuolar membrane, releasing the inner membrane and its contents (the autophagic body) into the vacuolar lumen. Cargo degradation is carried out by resident acid hydrolases, before membrane-bound effluxers such as Atg22p return components to the cytosol [14]. In order to monitor autophagy, Atg8p, a core Atg protein of ubiquitin-like function, is often used as a marker. This protein (the yeast homologue of mammalian LC3) is crucial to the formation of the AP through its conjugation to phosphatidylethanolamine and mediation of membrane-tethering events [15]. Atg8p serves as a useful marker of macroautophagy because this protein remains associated with the AP membrane, while other elements of the core Atg machinery only interact transiently [15].

Uptake of material into the vacuole lumen has also been shown to occur directly at the vacuolar membrane in a process known as microautophagy (Figure 1(b)) [16]. This process, observed in yeast, is characterised by the formation of an invagination at the vacuolar membrane, where cytosolic contents can be captured. The invagination grows into a tube-like structure within the vacuole that eventually pinches off from the vacuolar membrane, encapsulating the cytoplasmic contents within a single-membrane structure now located within the vacuolar lumen [16]. This microautophagic vesicle, along with its cargo, is subsequently degraded, apparently in the same manner as the autophagic body arising from the delivery of an AP to the vacuole. The molecular details of microautophagy and its physiological role largely remain unclear. Further work is required to characterise this phenomenon and whether observations made in yeast are relevant to mammalian cells [17].

As the relevance of autophagy as a fundamental cellular process has become increasingly evident in the literature, much research has focussed on selective manifestations of autophagy. The cytoplasm-to-vacuole targeting (Cvt) pathway, a selective application of the autophagy machinery uncovered early in yeast autophagy research, delivers at least two hydrolases (aminopeptidase I and *α*-mannosidase) to the vacuole, where they are processed to a mature, enzymatically active form [18]. This demonstrates that components of the autophagy machinery can be applied to biosynthetic pathways in addition to the canonical catabolic processes of intracellular turnover. In addition to the vacuolar delivery of aggregated molecules such as hydrolases, the formation of an AP allows the sequestration of a range of cellular materials, from soluble proteins to whole organelles. For example, the selective autophagic removal of peroxisomes (pexophagy, [19]), endoplasmic reticulum (reticulophagy, [20]), ribosomes (ribophagy, [21]), and mitochondria (mitophagy, [22–24]) has been described to date, while parts of the nucleus are also degraded by piecemeal microautophagy of the nucleus (PMN) at an early stage, and late nucleophagy (LN) following prolonged nutrient stress [25–27]. Even invading pathogens such as viruses [28] and bacteria [29] can be eliminated in higher eukaryotes through autophagic processes, collectively termed xenophagy. In *S. cerevisiae*, all types of selective autophagy identified to date (with the exception of LN) require the function of Atg11p, a protein that is thought to act as a scaffold or adaptor protein that brings the core *ATG* machinery into contact with targets selected for degradation [30, 31].

Mitophagy has recently become the subject of much scientific interest. This is due in part to the central role of this organelle in various cellular processes, as well as the association of mitochondrial dysfunction with pathological conditions in humans such as the neurodegenerative Alzheimer's and Parkinson's diseases [32–34]. The inherently dynamic mitochondrial network, which continuously undergoes fission and fusion events, is essential for eukaryotic life as the site for the provision of vast amounts of ATP [35]. However, as they age and accrue damage, mitochondria also present a potential challenge to cells through the leaking of excess reactive oxygen species (ROS) and other molecules, such as the proapoptotic protein cytochrome *c*, causing diverse cellular pathologies [36]. Mitophagy, working in concert with other degradative systems [37], serves as the primary means of eliminating those mitochondria that are damaged or surplus to requirements. As a selective manifestation of autophagy, mitophagy employs the core autophagy machinery together with Atg11p [38] and several other gene products identified in recent research (Figures 1(c) and 1(d)) [39]. While proteins involved in mitophagy are diverse in structure and function, all cooperate to bring mitochondria destined for degradation into contact with the core autophagic machinery, thereby playing an important role in linking mitochondrial stress signals to autophagy. As we are still not able to completely describe the mechanism and regulation of mitophagy using the evidence collected so far, it is highly likely that further molecular components are yet to be identified.

In yeast, several genes have been associated with mitophagy. A summary of the key findings in yeast mitophagy research since the first study describing mitochondria within autophagic bodies [40] is provided in Table 1. Through investigation of deletion strain phenotypes, the *UTH1*, *AUP1*, *ATG32,* and *ATG33* genes have been directly implicated in mitophagy. *UTH1 *encodes a 37 kDa SUN-family protein that localises to the outer mitochondrial membrane (OMM) and the cell wall [41]. This protein, which has previously been implicated in the maintenance of cell wall integrity, was shown to confer increased life span during nitrogen starvation (N-starvation) in a Δ*uth1* deletion strain and was not essential for macroautophagy. In a subsequent study, the same group demonstrated an early phase of mitophagy induced by N-starvation, which involves the sequestration of mitochondria directly by the vacuole, as observed by electron microscopy (EM). This suggests mitophagy can occur by a microautophagic mechanism, termed micromitophagy (Figure 1(d)) [42]. “Normal” macromitophagy, for which Uth1p is not required, follows this at a later stage.

In contrast, cells deleted for *AUP1*, which encodes a 49 kDa mitochondrial protein phosphatase, show perturbed mitophagy in long-term stationary-phase cultures and are characterised by decreased cell life span under these conditions [43]. A subsequent study linked *AUP1 *function to the retrograde signalling (RTG) pathway, perturbation of which by deletion of the *RTG3* gene resulted in a defective mitophagy phenotype [44].

Two recently reported whole-genome screens for genes involved in mitophagy both identified the gene encoding Atg32p, a single-pass mitochondrial outer membrane protein with a predicted molecular mass of 59 kDa [45, 46], as being required for mitophagy. This protein is able to interact with both Atg8p (a core autophagy protein essential for the biosynthesis of APs [47]) and Atg11p (essential for all forms of selective autophagy described to date), linking mitochondria marked for degradation with the core autophagic machinery (Figure 1(c)). While the mechanism by which mitochondria are selected for autophagy remains poorly understood, it is hypothesised that Atg33p, which is believed to be a mitochondrial outer membrane protein, is able to report mitochondrial stress to Atg32p, especially during post-log (stationary) phase of growth [48]. What triggers Atg33p to relay this mitophagy-inducing signal remains unclear.

Proteins specific to mitophagy function in a sequential and controlled process of mitochondrial degradation. This tight control reflects the two-fold role of mitophagy in cells: it is involved in maintenance of mitochondrial homoeostasis (i.e., the dynamic maintenance of the functional stability of mitochondria), and as a response to stress (the physical and chemical demands of a particular environment) [49]. This review focuses on mitophagy as a response to stresses both intrinsic and extrinsic to the mitochondrion. Following a brief overview of the signalling pathways known to be involved in the regulation of this process, we identify and discuss discrepancies in the literature with reference to the diversity of mitochondrial stresses, and how the cell coordinates its response to bring about mitophagy. We conclude that these discrepancies are indicative of a complex integration of the basic mechanism of mitophagy into the cellular milieu, and that experimental conditions employed in studies of mitophagy must be considered to fully grasp the role of this process within the cell.

## 3. Mitophagy as a Response to Stress

**Figure 2:**
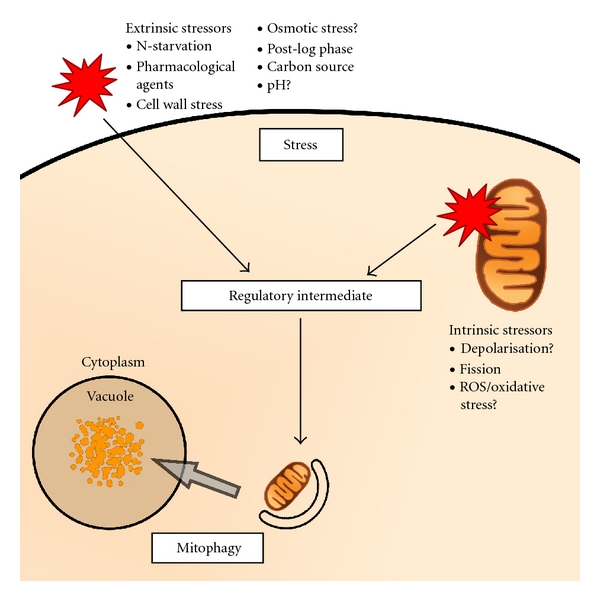
Mitophagy as a response to stress. Stress signals (red), arising from outside (extrinsic) or within (intrinsic) the mitochondrion, interact with regulatory intermediates in the cell. These intermediates coordinate the cell's response to these stresses, in this case promoting the removal of excess or dangerous mitochondria. As a consequence, mitochondria are then removed by an autophagic mechanism, mitophagy. Hypothesised, but as yet unconfirmed, stressors are indicated by “?”.

Mitochondria play a fundamental role in cellular metabolism through the supply of energy as ATP. For the cell, the maintenance of a “balance” between healthy mitochondria and those that are damaged or dangerous is essential in order to ensure the most efficient production of energy. This is a highly dynamic process requiring the cell's constant adaptation to changes in conditions within and outside of the cell. While any definition is necessarily problematic, for the purposes of this discussion, we define conditions that shift mitochondrial homeostasis in a direction favouring mitochondrial removal as stressors. Accordingly, cells that are subjected to such conditions are described as being in a state of, or exposed to, stress. Stress and stressors constitute the first step of mitophagy induction in which a stress signal is directed to the mitophagic response through a regulatory or signalling intermediate (Figure 2). It is important to recognise that stress, signalling/regulation, and mitophagy are overlapping terms in a continuum of controlled mitochondrial degradation. For the purposes of this discussion, we categorise stress as being either *intrinsic* (i.e., originating from within the mitochondrion itself) or *extrinsic* (arising anywhere outside of mitochondria, including within other parts of the cell) see Figure 2.

### 3.1. Intrinsic Stress

Much research has focussed on the role of the mitochondrion in triggering its own removal by mitophagy. Stresses originating from within mitochondria are often associated with mitochondrial damage, which affects the organelle's ability to produce energy efficiently without the release of excess ROS. In most physiologically relevant cases, mitochondrial damage is accompanied by the depolarisation of this organelle, or loss of the mitochondrial membrane potential (ΔΨ_*m*_), which is essential for generation of ATP. The interest in mitochondrial damage as a trigger of mitophagy has been promoted by the finding that in mammalian cells, mitochondrial damage is a precursor to mitophagic degradation by proteins implicated in Parkinson's disease [56].

### 3.2. Depolarisation, Damage, and Dynamics of Mitochondria

While the importance of mitochondrial depolarisation and fragmentation to mitophagy are well established in mammalian cells, studies in yeast have yielded conflicting results. An early report suggests a role in mitophagy for *MDM38*, which encodes a membrane protein involved in K^+^/H^+^ exchange and protein export. This study indicates that yeast cells deleted for this gene are characterised by swollen and fragmented mitochondria that are targeted for removal by mitophagy [52]. Deletion of *MDM38 *also results in abnormal mitochondrial morphology, with a collapse of the organelle cristae. These observations are consistent with the results of another study indicating that deletion of *FMC1*, which is required for ATP synthase assembly at high temperature, results in cells showing aggregation of ATP synthase F_1_ catalytic subunits in the mitochondrial matrix, and evidence of mitophagy [51]. In contrast, a recent study assessing various fission-related yeast genes concluded that fission is not required for mitophagy and that fission is neither a precursor to, nor an inducer of, mitophagy [55]. Defective mitophagy in a Δ*fis1* mutant was attributed not to the role of Fis1p in fission, but an indirect disruption of the gene *WHI2*, which encodes a protein involved in the general stress response. Furthermore, while uncoupling agents such as carbonyl cyanide *m*-chlorophenyl hydrazine (CCCP) are able to induce mitophagy in mammalian cells, this is not the case in yeast [41, 46, 55]. The two recent, genome-wide screens for genes involved in mitophagy also yielded conflicting results in terms of fission- and fusion-related proteins. According to data provided by Kanki et al., deletion of *DNM1*, encoding the important mitochondrial fission protein Dnm1p, significantly perturbs mitophagy [48], whereas Okamoto et al. did not detect the perturbation of mitophagy in strains deleted for any of the fission-related genes [45]. Clearly, further work is required to clarify these discrepancies.

### 3.3. Oxidative Stress and ROS

Oxidative stress often arises from within the mitochondrion, most commonly in the form of ROS. By definition, ROS are highly reactive molecules comprising oxygen, with reactivity attributable to the oxidising ability of unpaired valence electrons [57]. The healthy cell takes advantage of this useful oxidising property: controlled amounts of ROS play an important role in cell signalling and other redox-dependent processes [58]. However, ROS can also be hazardous to cells as they rapidly oxidise cellular components including amino acids, nucleic acids, and lipids. If ROS are allowed to accumulate, the consequences for the cell are dire and can result in death. A major source of ROS in cells is the mitochondrion, where ROS are a by-product of oxidative phosphorylation [59]. Under normal conditions, cells have adapted to cope with the production of ROS, eliminating these dangerous molecules by a range of antioxidant defences, such as superoxide dismutase, catalase, and glutathione [57].

The role of ROS as a regulator or inducer of mitophagy is not obvious given the available data, but work has provided several clues that allow preliminary speculation. As mitochondria produce the majority of intracellular ROS, imbalance of ROS levels and the resulting oxidative stress within these organelles is an attractive candidate for an inducer of mitophagy. ROS have been associated with nonselective macroautophagy in many recent studies in mammals [60, 61], as well as yeast nonselective autophagy [62]. However, in yeast, there is little evidence to suggest that ROS are able to induce mitophagy through any direct interaction with a component of the mitophagy machinery. A recent study by Suzuki et al. investigating cell death in autophagy-deficient yeast cells provided evidence that ROS accumulates in mutant strains lacking expression of certain *ATG *genes during nitrogen-starvation [63]. The implication here is that *ATG *genes play a role in the elimination of ROS-producing organelles, potentially by mitophagy. However, this is apparently due to the inability of these strains to upregulate expression ROS scavengers and respiratory chain components, rather than a direct inability of excess ROS-producing organelles to be removed by autophagy.

In a separate study, rapamycin (an established inducer of autophagy and mitophagy) was reported to reduce the cellular load of ROS in yeast cells, an effect attributed to an increase in mitophagy through target of rapamycin (TOR) signalling [64]. As removal of mitochondria by mitophagy reduces ROS load, these observations suggest that mitophagy is able to target ROS-producing organelles, although a direct relationship between ROS and mitophagy is not found. The fact that mitophagy could be further induced by rapamycin indicates that ROS alone in this case were not sufficient to induce complete mitophagy of these damaged organelles. At present, therefore, it seems that there is little evidence to support a direct role for ROS in mitophagy induction in yeast, although as important redox signalling molecules they are most likely indirectly involved.

### 3.4. Extrinsic Stress

In addition to the intrinsic factors dictating mitochondrial fate, a number of extramitochondrial stresses must be considered when considering mitophagy. Examples of such extrinsic stresses are pharmacological agents and the environmental conditions experienced by cells. Much research has investigated the link between extrinsic stresses and mitophagy in *S. cerevisiae*. Indeed, while mammalian cells generally exist within relatively stable tissue environments, unicellular organisms such as yeasts are often exposed to stressful environmental conditions. Such conditions can also be encountered by mammalian cells in unusual but clinically relevant circumstances, an example of which is the environment within tumours, where uncontrolled growth restricts the normal supply of nutrients to these extremely metabolically active cells [65]. The ease with which yeast cells can be exposed to environmental stress in the laboratory facilitates studies in this model organism. Yeast require a source of both nitrogen and carbon to survive and proliferate [66], and omission of either of these from the culture medium constitutes a starvation condition.

### 3.5. Nitrogen-Starvation

Nitrogen-starvation (N-starvation), in particular, is a well-established means of inducing both autophagy and mitophagy in yeast [40, 42, 48, 54, 63, 67]. This is achieved by transferring cells from a nitrogen-rich preculture medium to a medium omitting all sources of nitrogen, including amino acids. Such media can be supplemented with any source of carbon. Yeasts subjected to this form of stress cease proliferation and immediately activate autophagy in order to supply nitrogen for essential cellular processes [5]. Mitophagy is also induced by N-starvation, although the extent to which mitophagy is induced appears to depend on the particular carbon source available for cellular metabolism. For example, when yeast grown in rich media requiring mitochondrial function are subjected to N-starvation in media supplemented with sources of carbon that yeast can ferment by glycolysis (providing ATP independently of mitochondria, such as glucose), N-starvation results in mitochondrial turnover that is rapid and extensive [38, 41, 48, 53, 54]. Little mitophagy appears to occur, however, when yeast cells are subjected to N-starvation in medium supplemented with respiratory sources of carbon (requiring mitochondrial function to generate ATP, such as lactate) [38]. However, evidence discussed below suggests that exceptions to these rules are apparent—even when comparing carbon sources utilised by the same metabolic pathway, the extent and rate of mitophagy are not consistent. In any event, induction of mitophagy in this case can be attributed to TOR signalling (discussed below), although the mechanism by which mitophagy is suppressed in media containing a respiratory carbon source remains to be clarified.

### 3.6. Post-Log (Stationary) Phase of Growth

A form of stress observed to induce both mitophagy and autophagy is arguably the most natural condition of post-log (also referred to as “stationary”) phase of growth. In their natural environment, yeasts, being immotile microorganisms, rapidly utilise any available nutrients to proliferate. After these nutrients have been exhausted, yeasts enter a quiescent state of low metabolic activity and may undergo sporulation in order to survive until more favourable conditions for growth are encountered once again. Mimicking these conditions in the laboratory by culturing yeast in nutrient-rich medium for extended periods induces both autophagy and mitophagy. Conditions ranging from 3 days [38] to 5 days [43, 68] of growth in nutrient-rich medium supplemented with respiratory or fermentative carbon source have been reported as strageies to induce and study mitophagy. While the level of mitophagy observed in stationary phase cultures is extensive and represents a physiologically relevant and natural response, it is difficult to determine the exact source of stress; whether mitophagy is induced by depletion of nutrients, the buildup of waste products or a combination of factors has not been determined.

Culture to stationary phase was employed in studies identifying the role of Aup1p in mitophagy [43, 44], while Okamoto et al. and Kanki et al. both performed genome-wide screens for mitophagy-related genes under stationary phase conditions, identifying the mitophagy-specific protein Atg32p [45, 48]. Indeed, in the genome-wide screens carried out and reported by Kanki et al., Atg33p was shown to be involved in stationary phase mitophagy, but not in mitophagy triggered by N-starvation [48]. It would, therefore, seem that, while it is difficult to attribute mitophagy to any particular stress during stationary phase, other factors apart from the exhaustion of nitrogen supply are at play under these conditions. In spite of these difficulties, the relevance of stationary phase as a naturally encountered stress is clearly important.

### 3.7. pH

pH-stress has been linked with autophagy, although its role in mitophagy is not as clear. A long-standing question of why *ATG* mutants die prematurely in comparison to wild-type strains when cultured in starvation media was recently addressed in a study finding that certain *ATG* mutants are extremely sensitive to low pH (around pH 3) in unbuffered starvation culture medium [63]. Perhaps unexpectedly, this effect is apparently due to defective mitochondrial respiration. While this might suggest the disruption of mitophagy-mediated quality control over the mitochondrion, comparison of respiratory function in a Δ*atg32* (mitophagy-deficient) strain and numerous autophagy-deficient strains indicates that perturbation of nonselective autophagy and not mitophagy is responsible for this phenomenon. However, methods of inducing mitophagy may be accompanied by changes in pH, and it is important to keep an open mind to the possible role of pH in mitophagy.

### 3.8. Pharmacological Agents

A number of pharmacological agents are able to induce autophagy and mitophagy, thereby acting as mitophagy-inducing stressors. These pharmacological agents have diverse effects on cells, but usually act through the manipulation of cellular signalling and regulatory pathways that control autophagy. A key pharmacological agent commonly used in the field is rapamycin, which induces autophagy and mitophagy through the inhibition of TOR signalling [69, 70], and other agents such as CCCP (discussed above) and oligomycin (an inhibitor of ATP synthase and oxidative phosphorylation) have also been used in studies of mitophagy [41, 55]. While these treatments are useful in studies that interrogate specific mechanistic and regulatory questions, they are artificial in action and do not generally represent naturally occurring conditions. For this reason, pharmacological agents do not necessarily replicate natural changes in the regulatory networks of cells, and, therefore, may not elicit natural autophagic responses of physiological relevance.

### 3.9. Osmotic Stress

Osmotic stress has been associated with autophagy relatively recently, and the role of osmolarity in mitophagy requires further investigation. The osmoregulatory protein Hog1p, which functions in the MAPK pathway (discussed below), has been implicated in macroautophagy [71]. Deletion of *HOG1* resulted in reduced autophagy under conditions of hypo- or hyperosmotic stress, indicating that *HOG1* is important in the coordination of autophagy in response to osmotic stress. While Hog1p and other MAPK proteins have been implicated in mitophagy (discussed below), the deletion of the *HOG1* gene was found to result in the most severe perturbation of mitophagy. While MAPK is a key signalling pathway in the cell that responds to a range of stresses, these phenotypes may suggest that the osmotic status of the cell has some bearing on mitochondrial turnover.

## 4. Regulation of Mitophagy

As the mechanistic details of mitophagy and types of stress that induce the process become better understood, attention has turned to the cellular regulatory pathways that control mitophagy. It is important to note at the outset that our understanding of how autophagy and mitophagy are regulated is very much in its infancy. Fully characterising the regulation of autophagy and mitophagy, and indeed the question of how autophagy and mitophagy are integrated into the complex systems of cellular signalling, remain important challenges in the field. These questions are particularly important as the clinical implications of our knowledge become increasingly relevant.

The regulation of autophagy, reviewed elsewhere in great detail [72–74], is beyond the scope of this article. Recent research, informed by advances in our understanding of autophagy regulation, has implicated several key regulatory pathways in the regulation of mitophagy. The most relevant of these are discussed below.

### 4.1. TOR Signalling

The TOR signalling pathway has long been known to play a role in the regulation of autophagy. TOR signalling is conserved in some form throughout all eukaryotes and is intricately involved in cell proliferation and metabolism through its regulation of many cellular responses to nutrient status [66, 75]. TOR is sensitive to rapamycin treatment, and a vast body of literature supports the role this signalling pathway plays in the sensing of nitrogen supply [76–78]. Accordingly, TOR signalling is particularly relevant to regimes inducing mitophagy through N-starvation. However, due to the central role, it plays in the cell, TOR signalling is implicated in many stress responses, both related and unrelated to mitophagy.

The central components of TOR signalling in yeast are the two TOR complexes (TORCs), TORC1 and TORC2 [75]. Both TORCs are a collection of proteins that include Tor, a PI3-like protein kinase, but only TORC1 is sensitive to rapamycin and coordinates cell growth in response to nutrient availability [69, 79]. Under conditions of nutrient availability, TORC1 is active, allowing transcription and biosynthesis of genes and proteins required for cellular growth. Under such conditions, autophagy is repressed through the hyperphosphorylation of Atg13p, a core Atg protein required for autophagy [80]. In nutrient-poor conditions, however, TORC1 is inactivated and Atg13p is able to participate in the induction of autophagy. It is this inhibition of TORC1 that makes rapamycin such a potent and commonly used inducer of autophagy ([81, 82]).

While it is well established that TOR signalling is important for nonselective autophagy [83], the relationship between TOR and mitophagy remains unclear. In an early study demonstrating *UTH1 *involvement in mitophagy, it was demonstrated that treatment of cells cultured in respiratory medium with rapamycin induced mitochondrial turnover, eventually causing cell death [41]. Another early report in mammalian cells provided evidence that mitophagy is suppressed by TOR activity (i.e., was induced following rapamycin treatment) [84]. A more recent study in yeast has reported that treatment with rapamycin is able to reduce ROS production in cells deficient in frataxin (a mitochondrial iron chaperone), possibly by stimulating the removal of damaged mitochondria by autophagy [64]. While there is currently little direct evidence of TOR involvement in mitophagy, the role that this pathway plays in nonselective autophagy and nitrogen sensing, in particular, suggests the need for further investigation.

### 4.2. Mitogen-Activated Protein Kinase (MAPK) Signalling

Results of recent studies in yeast illustrate the role in mitophagy of two MAPK proteins, including Hog1p, Slt2p, and additional proteins associated with Hog1p and Slt2p function, including Wsc1p, Ssk1p, Bck1p, Mkk1p, Mkk2p, Pbs2p, and Pck1p [54, 85]. The MAPK pathway is a highly conserved, broad-ranging signalling cascade involved in a variety of cellular processes. MAPK signalling is involved in a range of pathways [86] but can be separated into two categories according to their role in cell proliferation or the transduction of stress signals [87]. Hog1p and Slt2p, core components of two pathways comprising the MAPK proteins listed above, are both involved in the MAPK response to stress. While Hog1p has been implicated in the response of yeast cells to osmotic stress [88], Slt2p is important in responding to stress at the cell wall [89]. According to Mao et al. [54], the inhibition of mitophagy in both Δ*slt2 *and Δ*hog1* deletion strains (and strains deleted for associated genes listed above) is marked, but not complete, suggesting that other as yet unidentified regulatory pathways are involved in the control of mitophagy. Temporally distinct regulation of mitophagy by Hog1p and Slt2p pathways is observed following the onset of N-starvation when monitored by Western blot analysis, echoing a trend described in the analysis of *UTH1*-dependent mitophagy [42]. Interestingly, Uth1p is also known to be involved in cell wall biogenesis [90], which suggests another link between this protein and Slt2p, and Wsc1p, which Mao et al. identify as having an effect on mitophagy, is also involved in the maintenance of cell wall integrity. The authors further find that autophagic role of Hog1p pathway proteins appear to be limited to mitophagy, whereas Slt2p associated proteins are also involved in the regulation of pexophagy, raising the prospect of crosstalk between the regulatory systems of different selective autophagy pathways. Indeed, hyperosmotic stress alone is not able to induce mitophagy, suggesting complexity in MAPK regulation of mitophagy [85]. It would, therefore, seem that consideration of mitophagy regulation in isolation of other selective autophagy pathways is unlikely to provide a complete understanding of the process.

### 4.3. Reduction-Oxidation Chemistry (Redox)

Redox chemistry is known to participate in a range of regulatory systems (reviewed in [59]), and an increasing body of evidence supports a role for redox chemistry in mitophagy. The ongoing question of ROS involvement in mitophagy, which has important implications for cellular redox balance, is considered separately above. The direct role of redox in yeast mitophagy was recently described by Deffieu et al., who showed that glutathione, a key cellular moderator of redox state and antioxidant [91], is linked to mitophagy regulation [53]. In this study, *N*-acetyl-l-cysteine (NAC) was shown to have an inhibitory affect on mitophagy, while it had no affect on nonselective autophagy. This inhibitory effect was attributed to NAC-associated increases in glutathione levels, altering the redox state of the cell. The changes in glutathione levels were also shown to be *UTH1*-dependent, suggesting that different regulatory regimes might promote different phases of mitophagy. Another study indicates that treatment of cells with NAC suppresses the expression of Atg32p, which accordingly inhibits mitophagy, suggesting a direct link between the redox state of the cell and the mitophagy machinery [45]. The perturbation of redox homeostasis is inexorably linked to the health of mitochondria and thus should allow us to investigate the relationship between mitochondrial damage and mitophagy further.

### 4.4. The Retrograde Signalling Pathway

Mitochondria are able to elicit transcriptional responses from the nuclear genome through the retrograde signalling (RTG) pathway. The RTG pathway, which partially overlaps with TOR signalling, provides the mitochondrion with a means for reporting stresses and metabolic challenges to the nucleus. As a key player in mitochondrial homeostasis, mitophagy is also regulated by the RTG pathway. Aup1p, a mitochondria-localised protein phosphatase required for stationary-phase mitophagy [43, 44], regulates the phosphorylation of the RTG transcription factor Rtg3p, as well as its localisation to the nucleus, leading to the activation of RTG genes [44]. Like Aup1p, Rtg3p was then shown to be required for stationary-phase mitophagy, but not nonselective autophagy or the Cvt pathway, although the redundancy of these proteins' actions was not determined. It is interesting to note, however, that TOR signalling regulated the localisation and activity of Rtg3p (and Rtg1p, another RTG transcription factor), suggesting a link between nitrogen sensing and mitophagy [92]. These findings suggest that the mitochondrion is not simply a passive subject of mitophagy; rather, mitochondria appear to play an active role in the regulation of their own removal by mitophagy. This is particularly interesting considering the increasing recognition of the mitochondrion as an active participant in cellular signalling on a number of different levels [93].

The regulation of mitophagy in yeast cells remains unclear, and further research in this area will provide further clues about the role of mitophagy and indeed mitochondria in the cell. The potential role of the TOR and MAPK signalling pathways as regulators of mitophagy suggests its integration into cellular responses to nutrient and other important stress signals. The implications of other potential regulators of mitophagy, including redox and RTG signalling, are not so obvious and warrant further research.

## 5. Mitophagy Mechanism and Regulation: Contrasting Observations

While significant progress has been made in our understanding of mitophagy, results emerging from a number of different studies remain to be reconciled. It is likely that the contrasting observations apparent amongst different studies, most clearly evident in the different outputs of the recent genome-wide screens for genes related to mitophagy, reflect the complex cellular integration of a variety of signal inputs in response to diverse conditions.

A variety of assays to monitor mitophagy have been employed, as well as a range of growth conditions and means of inducing mitophagy. We now examine the effect that these different experimental approaches might have on the mitophagy phenotypes observed with reference to contrasting observations that have emerged in yeast mitophagy research.

### 5.1. Inducing and Monitoring Mitophagy

Before discussing individual results, it is important to consider several practical aspects of yeast cell culture and mitophagy induction in the context of experimental design. The selection of relevant experimental conditions is paramount in order to best test a hypothesis. In terms of mitophagy, it is difficult to know what conditions are most relevant for the identification of mitophagy-related genes, even without considering the physiological relevance of experimental conditions. Several features, in particular, have to be considered when designing experiments. Firstly, the source of carbon in the medium has important implications for yeast cell phenotypes. Supplementation with a carbon source favouring fermentative growth, such as glucose, suppresses respiration in yeast (known as glucose repression) and the mitochondria fail to fully mature into an extensive reticular network [94]. In contrast, supplementation with sources of carbon utilised by respiration promotes the maturation of mitochondria into a filamentous reticular network that can be visualised under appropriate conditions. As mentioned above, the source of carbon has also been shown to affect the extent of mitophagy in cells. Transcriptional profiles of yeast cells differ significantly when cultured in carbon sources that must be utilised by the same means of catabolism [66]. The source of carbon in the culture medium is, therefore, an important consideration when interpreting results.

The means of inducing mitophagy is another important consideration when investigating mitophagy mechanisms and regulation. There are four different strategies adopted by researchers to experimentally induce mitophagy: N-starvation, treatment with pharmacological agents, causing mitochondrial dysfunction, or culturing cells to post-log phase. We do not currently have a complete understanding of the mechanisms by which each of these conditions trigger mitophagy, apart from understanding, for example, that TOR signalling is involved in both N-starvation and in response to treatment with rapamycin. This is of particular import in the case of post-log phase mitophagy induction; while this condition is most likely to replicate conditions experienced by yeasts in the wild, it is also likely the most problematic condition in terms of the isolation of variables inducing mitophagy. There is, therefore, a tension apparent between “natural” conditions, which result in more general induction, and “artificial” conditions, which manipulate particular variables. The former has the advantage of physiological relevance, while the latter can address more specific biochemical questions.

**Figure 3:**
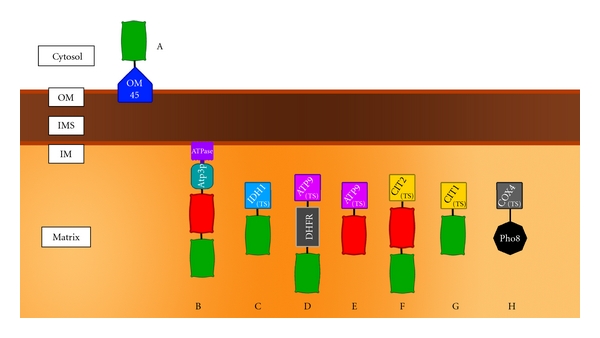
Biochemical probes for monitoring mitophagy in yeast. The localisation of probes within the different compartments of the mitochondrion is shown. A–G are fluorescence-based probes, while H is an enzymatic approach. (A) OM45-GFP is expressed from a chromosomal location in fusion with the endogenous OM protein OM45. GFP is exposed to the cytosol. (B) mt-Rosella II is an nonoligomerising biosensor comprising a red FP and pH-sensitive GFP expressed as a fusion to ATP3 from a genomic location (Lucarelli, May, Devenish and Prescott; unpublished). (C–H) Plasmid-derived combinations of FPs are targeted to the matrix space using different targeting sequences (TS) as follows: (C) isocitrate dehydrogenase, (D–E) F_0_ ATP synthase subunit c and (F–G) citrate synthase. (H) mito-Pho8 is a an acid phosphatase that is only active at vacuolar pH. When targeted to the matrix by a *COXIV* TS, the enzymatic activity provides a measure of mitophagy in strains disrupted for the endogenous *PHO8* and *PHO13* genes. Alternative targeting sequences allow targeting of probes to different compartments. OM = mitochondrial outer membrane, IMS = intermembrane space, IM = mitochondrial inner membrane, TS = targeting sequence.

The assay used to detect mitophagy is an additional experimental feature that requires consideration (reviewed in [70, 95, 96]). Assays are characterised by different sensitivities (detection thresholds) and, in general, are only as useful as the biological mechanism upon which they depend. The range of different assays employed to monitor mitophagy in yeast, which are represented in Figure 3, use different molecular strategies to detect mitophagy and produce different outputs. Fluorescent proteins (FPs) are often used to monitor mitophagy, either by directly observing changes in fluorescence signal (e.g., delivery to the vacuole) at the microscope, or through biochemical techniques such as enzymatic activity. Access to such assays represents an important means of verifying results but is also a potential source of variability. An example of this is found in the fluorescent protein- (FP-) based analyses. Probes currently in use are targeted to different compartments of the mitochondrion, expressed from either a chromosomal location or a plasmid, under the transcriptional control of different promoters and report mitophagy in different ways (Figure 3). These variables can all have an impact on the nature of the information reported by assays, as well as how the data they yield are interpreted. However, the range of data generated in studies employing different assays can be important to confirm experimental outcomes.

### 5.2. Differences Evident in the Literature and Experimental Conditions

There are several contrasting suggestions documented in Table 2. While separate studies have identified different proteins, these inconsistencies are not irreconcilable and may be attributed to differences in technical strategy. *UTH1 *was implicated in mitophagy by examining cells cultured first in lactate-supplemented medium that were shifted to N-starvation medium supplemented with either glucose or lactate [41, 42]. Under these conditions, mitophagy is observed from 2 hr after shifting to N-starvation. In contrast, the role of *AUP1* was determined in cells cultured to post-log phase of growth to induce mitophagy [43]. These genes are most likely involved predominantly in the mechanism of mitophagy induced in response to the particular experimental conditions employed in these studies. These findings exemplify the complexity of mitophagy in response to environmental cues. Indeed, such diversity in mechanism might not have been revealed without the use of different conditions to induce mitophagy.

As stated above, the physiological role of mitophagy in yeast is still not clearly understood. In 2005, Priault et al. analysed a temperature-sensitive Δ*fmc1* deletion strain, which is characterised by perturbation of both inner-membrane fusion and fission of the mitochondrial network at nonpermissive temperatures [51]. Microscopy and biochemical analyses indicated that mitochondrial morphology was severely perturbed under these conditions and that mitochondria lost ΔΨ_*m*_ before they were removed by mitophagy. Indeed, ΔΨ_*m*_ alone was found to be capable of mitophagy induction as recapitulation of respiratory incompetence in a wildtype strain was sufficient to induce mitophagy. This is in contrast to data provided by Nowikovsky et al. indicating that conditional deletion of *MDM38*, causing perturbation of mitochondrial morphology (in the form of extensive fission), osmotic swelling of the organelle, and loss of ΔΨ_*m*_, ultimately resulted in mitophagy [52]. These authors suggested that osmotic swelling and not alteration in ΔΨ_*m*_ was important for the induction of mitophagy. A subsequent study [55] employed a temperature-sensitive strain, *mgm1-5,* that shows defective inner membrane fusion, causing mitochondrial fragmentation at elevated temperature. Growth at elevated temperature was not sufficient to induce mitophagy, suggesting that mitochondrial depolarisation and fission are not linked to mitophagy. Indeed, CCCP treatment of yeast cells was not able to induce mitophagy, and blocking of mitochondrial fission did not induce mitochondrial degradation.

Interestingly, cells used in the study by Mendl et al. [55] were cultured in respiratory medium containing glycerol as carbon source, whereas Priault et al. [51] and Nowikovsky et al. [52] assess cells grown in fermentative medium containing glucose or galactose, respectively. This might suggest that as long as mitochondria are required to utilise the available carbon source, mitophagy is inhibited, even following a significant mitochondrial insult such as mitochondrial fragmentation. This is in line with observations made by Kanki et al. [38, 98] that a shift to N-starvation medium supplemented with a respiratory source of carbon is not sufficient to induce mitophagy. However, galactose (as used by Nowikovsky et al.) is known to not completely suppress mitochondrial function [99], and Kiššová et al. found in their 2007 study that mitophagy does occur in cells subjected to N-starvation supplemented with lactate, which is utilised by respiration [42]. Yeasts subjected to N-starvation in medium supplemented with ethanol as a respiratory carbon source undergo extensive mitophagy (May, Devenish and Prescott, unpublished results and [97]).

These observations provide evidence suggesting that the source of carbon is an important factor influencing mitophagy phenotypes, even when comparing carbon sources utilised by the same metabolic pathway. A more comprehensive analysis of the influence of culture conditions on mitophagy phenotype should provide an interesting perspective on the place of mitophagy in metabolic homeostasis.

### 5.3. Two Genome-Wide Screens for Genes Involved in Mitophagy

There are some intriguing differences in the results generated by the two yeast genome-wide screens for mitophagy genes performed in 2009 by Kanki et al. and Okamoto et al. [45, 48], who analysed 4667 and 5150 deletion strains, respectively, for mitophagy defects. Kanki et al. conducted the screen in several phases, initially screening all deletion strains grown to post-log phase (3 days) in medium supplemented with lactate. This phase detected 290 deletion strains that were not deleted for *ATG* genes and grew normally. In the second phase, deletion strains were cultured in nutrient-rich lactate medium before being shifted to glucose-supplemented N-starvation medium to induce mitophagy. In total, 65 deletion strains were characterised by abnormal mitophagy, of which 32 had a clear defect in mitophagy. Ultimately, 23 of these were identified as novel mutants not otherwise linked to mitophagy.

In contrast, Okamoto et al. cultured cells to post-log phase in medium supplemented with glycerol for 5 days before determining mitophagy in deletion strains. This method detected 53 genes that when deleted conferred a defective mitophagy phenotype. Of these, 35 non-*ATG* genes were reported, of which 23 are novel candidates for involvement in mitophagy. Interestingly, only eight novel genes were characterised as having an unequivocal mitophagy defect in both screens—many genes reported were not detected in the alternate screen.

There are several possible reasons why the outputs of the two screens were different. In both screens, deletion strains were cultured in respiratory medium, although Okamoto et al. cultured cells further into post-log phase before analysing them for evidence of mitophagy. The use of N-starvation in the second phase of the Kanki et al. screen is a potential point of contrast between the two screens that may account for the different outcomes. However, there is little correlation in the identity of genes detected in the first phase of the Kanki et al. screen and the Okamoto et al. screen. Accordingly, the likely explanations are that either the probes used to detect mitophagy do not report the process with the same efficiency, the differences in culture length into post-log phase affect the type of mitophagy executed, or that the type of respiratory carbon source has an influence over mitophagy regulation or mechanism.

The fluorescent probes used in these studies differ in two ways: their mode of expression and their targeting to the mitochondrion (Figure 3 and discussed in [70]). Kanki et al. adopted an OM45-GFP probe that is encoded by a gene cassette integrated into the nuclear genome under expression control of the native OM45 promoter. OM45 localises to the mitochondrial outer membrane and is exposed to the cytosol. Okamoto et al. used a plasmid-borne gene cassette encoding GFP fused to dihydrofolate reductase (DHFR) and an ATP synthase subunit 9 targeting sequence, which delivers the probe to the mitochondrial matrix. Studies in mammalian cells have shown that the mitochondrial outer membrane can be delivered to other cellular compartments, such as the peroxisome [100], and that mitochondria can supply membranes during the membrane expansion step of AP formation [12]. The outer membrane may, therefore, be processed differently to the matrix during mitophagy. OM45-GFP has previously proven to be a reliable indicator of mitophagy in yeast [38, 98], although the behaviour and targeting of the probe may change under different conditions. However, it seems most likely that culture conditions employed by the two screens are responsible for the observed differences in mitophagy phenotype. Thus, the additional time spent in post-log phase by cells assessed by Okamoto et al., or the carbon source itself, should be considered as factors potentially influencing the course of mitophagy.

It is noteworthy that both genome-wide screens failed to retrieve a number of genes, including *AUP1*, *UTH1*, *MDM38*, *RTG3,* or *WHI2*, that are reported to play a role in mitophagy in other studies. For most genes, this can be attributed to the differences in growth medium (carbon source) and the means by which mitophagy was induced in these studies in comparison to the genome-wide screens. *AUP1* and *RTG3* are the exceptions here, as strains deleted for these genes were demonstrated by Journo et al. [44] to be defective for mitophagy under virtually the same conditions as those employed by Kanki et al. [48]. In addition, the screen performed by Kanki et al. found a slight mitophagy defect in a strain deleted for *FMC1*, whereas Priault et al. [51] found that deletion of this gene and the associated mitochondrial damage incurred induced mitophagy. This may be due to the use of a different background strain of yeast—both Kanki et al. and Okamoto et al. used the same strain of yeast (although different mating types), while Journo et al. used a number of other strains of different genetic backgrounds.

In light of these themes, it is interesting that the two signalling pathways implicated in the regulation of mitophagy in yeast thus far, MAPK and redox (by glutathione), were detected by separate groups culturing cells under similar conditions [53, 54]. This suggests that even amongst cells exposed to similar stresses, regulation is complex and requires the coordination of different signalling pathways. Indeed, the genes detected in the genome-wide screens are involved in a very broad range of processes in the cell, suggesting that mitophagy is a well-integrated and fundamental process of cellular life. Untangling the complexity of mitophagy through comprehensive analyses of different conditions promises to enhance our understanding of this intriguing process.

### 5.4. Differences between Mitophagy in Mammalian and Yeast Cells

While mammalian cells are not the focus of this review, it is important to consider some of the apparent differences when comparing mitophagy in yeast and mammalian cells. Yeast is considered to be an important model for studying fundamental biological cellular processes including autophagy. Ultimately, such a discussion also helps us to understand the place and appropriateness of yeast as a model of mammalian cells.

In contrast to nonselective autophagy, it appears that the mitophagy mechanism in mammalian cells is different to that in yeast. Research carried out in mammalian cells has uncovered two mechanisms of mitophagy. The first, which is thought to be involved in mitochondrial quality control, requires the OM-localised Ser/Thr kinase PINK1, which detects a stress signal [101]. PINK1 then binds Parkin, a cytosolic ubiquitin ligase, which then ubiquitinates target proteins on the mitochondrion [102]. The target for ubiquitination and the implications of this process are not understood, but mitochondria marked in this way are subsequently degraded by mitophagy. Importantly, the PINK1-Parkin system is strongly linked to Parkinson's disease: a loss-of-function mutation in PARKIN is the most common mutation associated with the early onset form of the disease [103]. The second form of mitophagy encountered in mammalian cells, NIX-dependent mitophagy, is associated with reticulocyte maturation [104]. The Bcl-2 family protein NIX (NIP3-like protein X) interacts directly with LC3, the mammalian equivalent of Atg8p, facilitating mitophagy. NIX has been associated exclusively with the elimination of mitochondria from maturing reticulocytes and is dramatically upregulated in these cells immediately before the entire mitochondrial population is degraded by mitophagy, although a recent study has questioned whether NIX is essential for removal of mitochondria from reticulocytes [105]. It is also important to note that a mammalian homologue of Atg32p has not yet been identified in mammalian cells. Further research, however, is likely to uncover more molecular components in mammalian cells.

Beyond the mechanism of mitophagy, there also appear to be differences in the stressors that can induce mitophagy in yeast and mammalian cells. As discussed above, studies assessing whether membrane depolarisation acts as a precursor to mitophagy in yeast cells have provided inconsistent conclusions. In mammalian cells, however, depolarisation is closely associated with mitophagy. The first indications that depolarisation of mitochondria is linked to mitophagy were made by Elmore et al. [106] who illustrated that the mitochondrial permeability transition, a pathological state characterised by an increased permeability of mitochondria to small molecules, precedes mitochondrial autophagy in mammalian cells. Subsequent research has further characterised this phenomenon in mammalian cells with regard to the role of PINK1 and Parkin. More recently, it has been demonstrated that mitochondria characterised by reduced ΔΨ_*m*_ are more likely to be separated from the intracellular population by fission events and that these depolarised organelles are unlikely to re-fuse [107]. These isolated mitochondria are more likely to be removed by mitophagy, supporting the hypothesis that mitochondrial dynamics and mitophagy coordinate to ensure the quality of a cell's mitochondrial population. Narendra et al. [56] subsequently demonstrated that Parkin is recruited in a selective manner to depolarised mitochondria and that Parkin localisation is essential for turnover by mitophagy. Interestingly, Amo et al. [108] found that swollen mitochondria and loss of ΔΨ_*m*_ evident in PINK1^−/−^ MEFs, which results in fragmentation and increased mitophagy, are due to disturbances in respiratory chain function. This result, which echoes the Suzuki et al. study of pH-effects on yeast mitophagy [63], suggests that permeabilisation may be a consequence rather than a cause of damage in this case. Interestingly, Dagda et al. recently demonstrated in mammalian cells that localisation of PKA, an upstream modulator of TORC, to the mitochondrial outer membrane prevents mitophagy in PINK1-deficient mammalian cells [109]. In summary, unlike mitophagy in yeast, depolarisation is a well-established precursor to mitophagy in mammalian cells.

While the influence of fission and fusion events on mitophagy is contentious in the yeast literature, the role of mitochondrial dynamics in mitophagy is well-established in mammalian cells (reviewed in [110]). In addition to the important contribution made by Twig et al. [107], other studies have supported the importance of fission and fusion as a means of mitochondrial quality control. Evidence suggests that knockdown of PINK1 results in mitochondrial fission and mitophagy [101], while another study demonstrates that PINK1 and Parkin ubiquitinate and subsequently cause the degradation of mitofusins (proteins involved in fusion events) on damaged mitochondria, promoting their isolation from the healthy mitochondria network [111]. Müller and Reichert [112] have speculated that fission and fusion may still play a role in basal mitophagy in yeast but that the level of such mitophagy may be too low to detect. This clear distinction between the effect of mitochondrial dynamics in yeast and mammalian cells may reflect a shift in the emphasis of mammalian mitophagy from the yeast-like adaptation to starvation to basal, maintenance mitophagy.

Although still in its early stages, preliminary work interrogating the relationship between ROS and mitophagy in mammalian cells suggests that the two are linked. Assessment of PINK1 knockdown by Dagda et al. revealed that ROS and H_2_O_2_ in particular are important upstream preconditions for effective mitochondrial fission and mitophagy [101]. Schertz-Schouval et al. have also shown that ROS oxidise the mammalian Atg4 protein at a cysteine residue, promoting AP formation and autophagy, as well as perturbing ΔΨ_*m*_ and causing mitochondrial permeability [113]. These data imply that ROS also play a role in redox regulation of autophagy and potentially mitophagy in mammalian cells. It will be interesting to determine whether ROS-induced APs are also involved in the removal of excess ROS-producing mitochondria. While the limited data available suggest that ROS are more relevant to mitophagy in mammalian cells, more evidence is required before we can begin to speculate on the meaning of these results. The optimisation of techniques used to monitor ROS should allow us to more confidently state the role in mitophagy of these molecules, which are notoriously difficult to follow due to their short-lived and reactive nature.

Differences in the implications of perturbed MAPK signalling for mitophagy are also observed between yeast and mammalian cells. As discussed above, there is strong evidence that specific stress-related MAPK proteins participate in mitophagy regulation in yeast [54]. Interestingly, the MAPK proteins most clearly implicated in mitophagy, the extracellular signal-related kinases (ERKs), are involved in cell proliferation rather than stress response [114]. Other MAPKs variously implicated in mammalian mitophagy and autophagy are known to coordinate stress responses (e.g., c-jun N-terminal kinase (JNK) and p38) [115]. However, the centrality of ERKs in mammalian mitophagy might also support the apparent emphasis on developmental and basal mitophagy in mammalian cells.

In light of such differences between mammalian and yeast mitophagy, it is important to reflect on the role of yeast in mitophagy research. As far as we can infer from the available data, there appear to be fundamental differences between mitophagy in yeast and mammalian cells, even at the level of basic mechanism. This is a reason to question the utility of yeast as a model of mammalian mitophagy. However, even though individual stressors, regulatory pathways or proteins involved in yeast mitophagy may differ from those in mammalian cells, yeasts still offer an opportunity to characterise an independent and highly responsive system of mitochondrial homeostasis. As discussed above, the differences evident in yeast cells and mammalian cells may be a reflection of the more complex role of mitophagy in multicellular organisms. Although mammalian cells exist within less stressful tissue environments, they are faced with greater developmental demands and must maintain their mitochondrial populations for a much longer lifespan. However, mammalian cells must still respond to mitophagy-inducing stress, especially under pathological conditions such as tumour growth and microbial invasion, which are of great clinical importance. The identification of such themes of physiological role and cellular context through yeast research offers a valuable base for studies in more complex mammalian cells. Understanding the relative importance of mitophagy in diverse aspects of cellular life, therefore, offers further depth in our understanding of fundamental cell biology.

## 6. Conclusion

Considerable advances in the basic mechanism of mitophagy have been described in both yeast and mammalian cells. However, our understanding of mitophagy is not complete, and accumulating evidence indicates that mitophagy is a complex and intricately regulated process within the cell. Even within the yeast literature, there is a significant number of contrasting observations concerning the mechanism and regulation of mitophagy. These differences, not yet fully reconciled, offer to provide researchers with a greater appreciation of the physiological relevance of mitophagy. The excistance of different mitophagy phenotypes observed under various conditions is itself evidence of an elaborate integration of mitophagy into the regulatory networks of the cell and strongly suggests that mitophagy plays an important role in the maintenance of cellular homeostasis. In order to deepen our understanding of this intriguing process, we contend that it is important to comprehensively assess, using a benchmark assay, the effect that individual changes in conditions such as carbon source and means of mitophagy induction have on mitophagy. With a greater understanding of how experimental variables affect mitophagy proteins and regulation, insights from yeast research promise to provide important information about the broader cellular context of this complex process, allowing us to better understand the significance of mitophagy.

## Figures and Tables

**Table 1:** Key findings in yeast mitophagy research.

Author	Year	Primary finding	Notes	Assay	Carbon source*	Mitophagy induction*	Reference
Takeshige et al.	1992	Mitochondria within autophagic bodies	First observation of mitochondrial autophagy	Light microscopy, EM	Glucose, glycerol	Shift to N-starvation medium (glucose or glycerol)	[40]

Campbell and Thorsness	1998	Observation of damage-induced mitophagy	Further early evidence of mitophagy	EM	Various (respiratory)	Mitochondrial damage through disruption of *YME1 *	[50]

Kiššová et al.	2004	*UTH1*	First mitophagy-specific gene identified	pGAL-CLbGFP (fluorescence microscopy)	Lactate & glucose	Shift to N-starvation medium (lactate and glucose) Rapamycin (0.2 *μ*g/mL)	[41]

Priault et al.	2005	Mitochondrial damage triggers mitophagy	Impairing ΔΨ_*m*_ results in preferential mitophagy of impaired mitochondria.	EM Pho8Δ60 (biochemical) Western (protein degradation)	Glucose (aerobic and anaerobic)	Used heat-sensitive Δ*fmc1* strain to precipitate mitochondrial damage.	[51]

Nowikovsky et al.	2007	*MDM38 *	Found osmotic swelling triggers, and fission and is required for mitophagy	pCS-G/RFP (“Rosella”, microscopy)	Galactose	Doxycyclin (5 *μ*g/mL, induced *MDM38* depletion and mitophagy)	[52]

Kiššová et al.	2007	*UTH1*	Description of selective mitophagy and “micromitophagy.”	EM	Lactate throughout	Shift to N-starvation medium (lactate)	[42]

Tal et al.	2007	*AUP1*	*AUP1* role in post-log phase mitophagy described	Western (aconitase degradation)	Glucose, lactate	Culture to post-log (glucose, lactate, up to 5 d)	[43]

Kanki and Klionsky	2008	*ATG11*	Further demonstration of selective mitophagy.	OM45-GFP, IDH1-GFP, ALP (biochemical)	Lactate	Shift to N-starvation medium (glucose)	[38]

Deffieu et al.	2009	Glutathione involvement	Indicates role of Redox in mitophagy induction	pGAL-CLbGFP (microscopy) EM	Lactate	Shift to N-starvation medium (glucose)	[53]

Kanki et al.	2009	*ATG33* (and 31 others)	Did not report *UTH1*, *MDM38*, *AUP1*, *RTG3* or *WHI2*. 8 reported genes overlap with Okamoto et al.	OM45-GFP (microscopy and western)	Lactate	Culture to post-log (lactate, 3 d) Shift to N-starvation medium (up to 6 h glucose)	[48]

Kanki et al.	2009	*ATG32*	Identified at same time as Okamoto et al.	OM45-GFP (microscopy & western)	Lactate	Culture to post-log (lactate, 3 d) Shift to N-starvation media (up to 6 h glucose)	[46]

Okamoto et al.	2009	*ATG32* (& 52 others, including some known autophagy genes)	Did not report *UTH1*, *MDM38*, *AUP1*, *RTG3*, *WHI2* or* ATG33*. 8 reported genes overlap with Kanki et al.	p416GPD-mtDHFR-GFP (microscopy)	Glycerol	Culture to post-log (glycerol, 5 d)	[45]

Journo et al.	2009	*RTG3*	Also found *RTG3* regulates *AUP1*.	Fluorescence microscopy & Western analyses IDP1-GFP (microscopy)	Lactate	Culture to post-log (lactate, 3 d)	[44]

Mao et al.	2011	*HOG1*, *SLT2 *	Shows MAPK signalling is involved in mitophagy in yeast	OM45-GFP(microscopy & western)	Lactate	Shift to N-starvation media (6 h, glucose) Culture to post-log (lactate)	[54]

Mendl et al.	2011	*WHI2 *	Found fission is not essential for mitophagy.	pRS313-mtDsRed.T4 (microscopy)	Glycerol	Rapamycin (1 *μ*M, 24 h, in DMSO)	[55]

*Where “*carbon source*” and “*mitophagy induction*” refer to conditions used to detect the primary finding.

GFP = green fluorescent protein, ΔΨ_*m*_ = mitochondrial membrane potential, MOM = mitochondrial outer membrane, EM = electron microscopy, MAPK = mitogen activated protein kinase, N-starvation = nitrogen-starvation.

**Table 2:** Contrasting observations in yeast mitophagy research.

Observation	Supporting studies	Contradicting studies
Depolarisation triggers mitophagy	[51, 52]	[55]
Fission precedes mitophagy	[52]	[55]
Mitophagy by microautophagy (micromitophagy)	[42]	
Stress and regulation of mitophagy		
* -TOR*	[41, 55]	
* -MAPK*	[54]	
* -Redox*	[53]	
* -RTG*	[44]	[45, 48]*
* -General stress*	[55]	
* -pH*		[63]
Proteins required for mitophagy		
* -Uth1p*	[41, 42]	[45, 48]*
* -Aup1p*	[43]	[45, 48]*
* -Atg32p*	[45, 48]	
* -Atg33p*	[48]	
Requirement of nonrespiratory medium to induce mitophagy under N-starvation	[38, 48]^†^	[42, 52, 97]

*These studies reported no evidence of involvement, but did not directly contradict the observation.

^†^In this study, limited mitophagy is demonstrated during lactate-supplemented N-starvation.
